# Co-Occurrence of *bla*_OXA-23_ in the Chromosome and Plasmid: Increased Fitness in Carbapenem-Resistant *Acinetobacter baumannii*

**DOI:** 10.3390/antibiotics10101196

**Published:** 2021-10-01

**Authors:** Zhiren Wang, Henan Li, Jiangang Zhang, Hui Wang

**Affiliations:** Department of Clinical Laboratory, Peking University People’s Hospital, Beijing 100044, China; 2011210279@pku.edu.cn (Z.W.); zjg@bjmu.edu.cn (J.Z.)

**Keywords:** carbapenem-resistant *Acinetobacter baumannii*, *bla*_OXA-23_, multiple copies, plasmid, fitness

## Abstract

This study aims to explore the co-occurrence of chromosomal and plasmid *bla*_OXA-23_ in carbapenem-resistant *A. baumannii* (CRAB) and its influence on phenotypes. A total of 11 CRAB isolates containing copies of *bla*_OXA-23_ on the chromosome and plasmid (CO), as well as 18 closely related isolates with *bla*_OXA-23_, located on either the chromosome or plasmid (SI), were selected for the determination of antibiotic susceptibility, virulence phenotype, and characteristic genomic differences. The co-occurrence of *bla*_OXA-23_ on the CRAB chromosome and plasmids did not enhance carbapenem resistance, but trimethoprim/sulfamethoxazole exhibited significantly reduced minimum inhibitory concentrations in CO. CO demonstrated a higher degree of fitness compared to SI. An increased biofilm formation ability and serum tolerance were also identified in CO, which may be associated with virulence genes, which include *csuD*, *entE*, *pgaA*, and *plc*. *bla*_OXA-23_-carrying transposons were found at different insertion sites on the chromosome. The most common site was AbaR-type genomic islands (50%). Two types of plasmids were found in CO. The co-occurrence of *bla*_OXA-23_ on the chromosome and a plasmid in CRAB had little effect on carbapenem susceptibility but was accompanied by increased fitness and virulence. Different origins and independent insertions of *bla*_OXA-23_-carrying transposons were identified in both the chromosomal and plasmid sequences.

## 1. Introduction

*Acinetobacter baumannii* is a Gram-negative, opportunistic, and nosocomial pathogen that causes hospital- and community-acquired infections. This pathogen is considered a global threat to public health because of the speed at which it develops resistance to antibiotics and because residual options for treating its infections are limited. A recent review reported a carbapenem-resistant *Acinetobacter baumannii* (CRAB) prevalence of about 80% in China [1]. Poor clinical outcomes have also been reported with CRAB infections [2]. 

Carbapenems are broad-spectrum antimicrobial agents. They are members of the β-lactam family that are active against most β-lactamase-producing organisms. Carbapenems are accepted as the first-line treatment for multidrug-resistant bacterial infections [3]. However, the increasing trend in the occurrence of CRAB indicates that last-resort treatments are increasingly ineffective. Many resistance mechanisms have been described for *A. baumannii* [3], including enhanced degradation of antibiotics by β-lactamases. OXA-type carbapenem-hydrolyzing class D β-lactamase, the universal β-lactamase in *A. baumannii*, plays a significant role in carbapenem resistance [4]. The *bla*_OXA-23_ gene is the most common determinant of carbapenem resistance in CRAB. Carbapenem resistance may also be attributed to modified porins, penicillin-binding proteins, and the resistance-nodulation-division (RND) family efflux system [3].

*A. baumannii* shows high genetic plasticity, allowing for the accumulation of resistance determinants and highlighting the correlation between the horizontal transfer of resistance genes through mobile genetic elements and the increasing incidence of CRAB [5]. This indicates that horizontal gene transfer may play an important role in *bla*_OXA-23_ dissemination. The ability of *bla*_OXA-23_ to spread is related to the combination of composite transposons. Presently, four transposons (Tn*2006*, Tn*2007*, Tn*2008*, and Tn*2009*) have been found to mediate the transfer of *bla*_OXA-23_ in CRAB [6]. Tn*2006* is the first and most frequently reported transposon harboring *bla*_OXA-23_. Tn*2009* has effectively contributed to the wide dissemination of *bla*_OXA-23_ in *Acinetobacter spp*. in China. However, whether the co-existence of *bla*_OXA-23_ on the chromosome and plasmid influences susceptibility to carbapenem remains unknown.

The co-occurrence of integrative and extra chromosomal mobile genetic elements in microbial populations is common. The conversion from plasmid to integrative and conjugative elements allows for the expansion of the organism’s host range [7]. At present, this co-occurrence phenomenon has been identified for the *mcr-1*, *bla*_AmpC_*,* and *tet*(X4) genes. *mcr-1* can co-exist in *Escherichia coli*, with triplicate copies on chromosomes and another copy encoded on a plasmid [8]. As opposed to lipopolysaccharide (LPS) modification and colistin resistance caused by a single copy of *mcr-1*, multiple copies contribute to the persistence of colistin resistance in the host isolate. Co-carriage of both chromosome- and plasmid-mediated *bla*_AmpC_ genes was observed in seven *Pseudomonas aeruginosa* isolates [9]. *tet*(X4) was identified on chromosomes and plasmids in one *E. coli* isolate [10]. For the carbapenem resistance gene *bla*_OXA-23_, there is a report of its multiplication on the chromosome that claims it does not enhance carbapenem resistance [11]. However, the situation about *bla*_OXA-23_ located on the chromosome and plasmid at the same time has not been attached. This study aimed to explore the co-existence of *bla*_OXA-23_ on both the chromosome and plasmid in CRAB and the degree to which multiplication enhances carbapenem resistance and persistence.

## 2. Results

### 2.1. Prevalence of the Co-Occurrence of bla_oxa-23_ on Chromosomes and Plasmids

**Figure 1:**
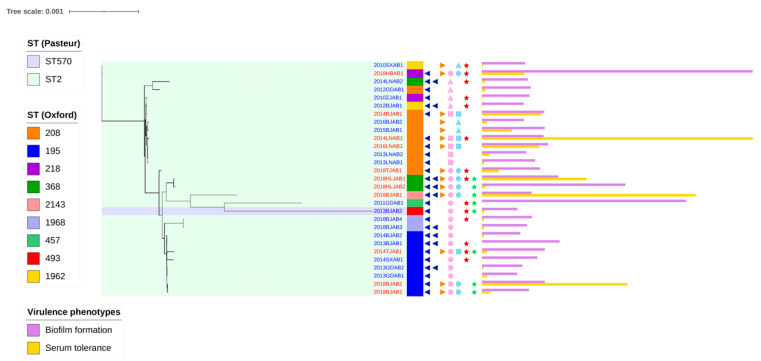
Phylogenetic tree of CO and SI isolates. The phylogenetic tree was constructed based on core single nucleotide variants of chromosomes. The internal colored ranges and the external colored stripes represented STs in Pasteur and Oxford typing schemes, respectively. The navy-blue left triangle and the orange right triangle indicated the location of *bla*_OXA-23_ in the chromosome and plasmid, respectively. The number of triangles indicated the number of copies of *bla*_OXA-23_. The circle, square, and upward triangle indicated that *bla*_OXA-23_ was flanked by Tn*2006*, Tn*2009,* or IS*Aba1,* while pink and azure classified gene location into chromosome or plasmid, respectively. The red and green stars represented minocycline and SXT resistant phenotypes. The purple and yellow bars represented the biofilm formation and serum tolerance virulence phenotypes.

Between 1999 and 2018, positive rates of CO isolates among our sequenced isolates showed an increasing trend over time. Before 2013, no CO isolates were identified. However, 3, 2, and 6 CO isolates were identified out of a total of 28 isolates (10.7%, 7.1%, and 21.4% positive rates, respectively) in 2014, 2016, and 2018, respectively. We determined the clonal relatedness of the 11 isolates using MLST (Table 1). All the isolates were identified as sequence type (ST) 2 using the Pasteur scheme. However, the Oxford scheme identified four ST208, three ST195, two ST368, one ST218, and one ST2143 isolate. In addition, aside from ST2143, all the other STs belonged to clone complex 208 (CC208). For a detailed analysis of isolate relationships and evolution, we developed a robust phylogeny based on single nucleotide variants present in the core regions of the genome that represent ancestral relationships between CO and SI isolate genomes (Figure 1). Phenotypic characteristics were also attached to the phylogenetic tree (as described below), which might reveal the relevance of evolutionary history and phenotypic changes in CO and SI isolates. The analysis indicated that isolates were roughly grouped into three distinct clades, with little evidence showing that CO evolved from current SI isolates, implying that co-occurrence of *bla*_OXA-23_ on chromosomes and plasmids occurred previously. We found no significant evolutionary relationships or genomic characteristic differences between CO and SI isolates. 

### 2.2. Resistance Gene Profiles and Correlation between Antibiotic Resistance and bla_OXA-23_ Location

We found all CO isolates were resistant to imipenem, meropenem, cefepime, ceftazidime, piperacillin/tazobactam (TZP), tetracyclines, doxycycline, ciprofloxacin, and levofloxacin. However, all CO isolates were sensitive to colistin. Susceptible rates to minocycline (18.2%), amikacin (18.2%), trimethoprim/sulfamethoxazole (SXT) (54.5%), and tigecycline (72.7%) increased progressively (Table 2).

To determine the correlation between *bla*_OXA-23_ co-occurrence and antibiotic resistance patterns, we performed antimicrobial susceptibility tests for SI isolates (Table S1). In addition, we used the unpaired Student’s t-test to compare the resistance profiles of CO and SI isolates (Figure S1). Among the 13 antibiotic agents listed above, only SXT showed significantly low MICs from the SI isolates (*p* < 0.05). The corrected Welch’s t-test results indicated significant differences in the MICs of TZP and imipenem (*p* < 0.05). TZP and imipenem exhibited lower and more discrete MIC distributions in SI isolates as compared to CO isolates. We observed a similar trend in the MICs of ceftazidime, cefepime, meropenem, and minocycline, but there were no significant differences in MIC values (*p* > 0.05). 

The MIC_50_ and MIC_90_ of minocycline in CO isolates were twice as high as those in the SI isolates. Moreover, the MIC_50_ and MIC_90_ of SXT in CO isolates were sixteen times and four times lower, respectively, than those in SI isolates (Table 2). We further analyzed the MICs of minocycline and SXT using the Chi-square test, which showed significantly different MIC values in CO and SI isolates for these two agents (*p* < 0.05). However, no significant differences were observed with the other antibiotics. 

**Figure 2:**
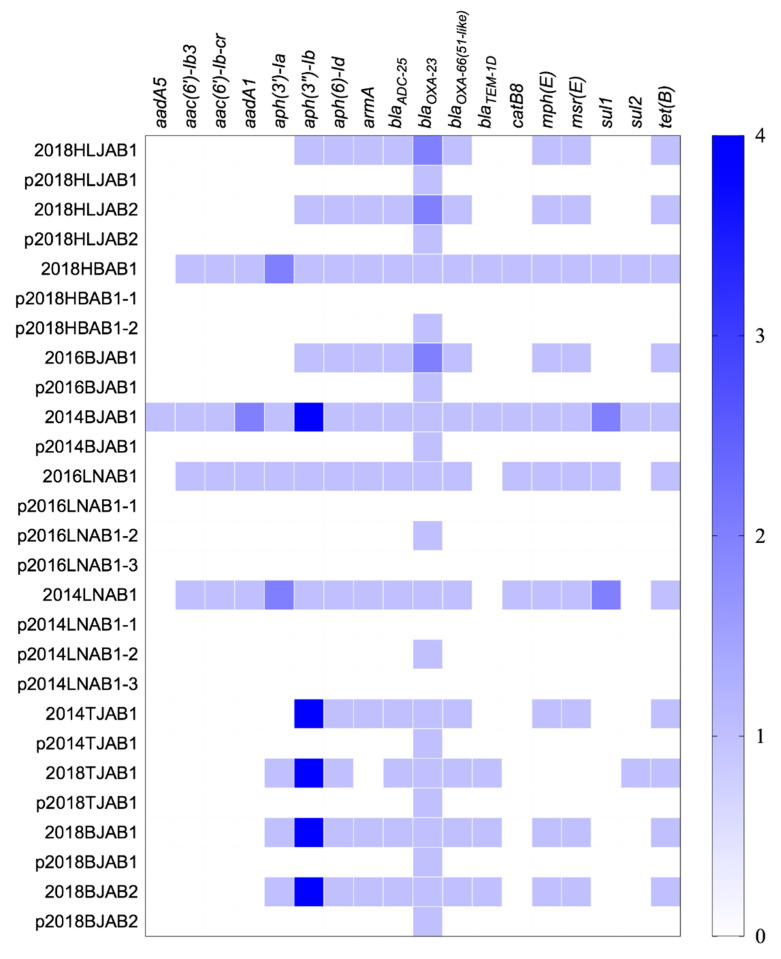
Distribution of antibiotic resistance genes in the chromosomes and plasmids of the 11 CO isolates. The color shade showed the copy numbers of genes.

The WGS of the 11 isolates revealed 18 known resistance genes on the chromosome, consistent with the reduced susceptibility to β-lactams (*bla*_OXA-23_ and *bla*_ADC-25_), macrolides (*mph*(E) and *msr*(E)), aminoglycosides (*armA*, *aph(3’’)-Ib,* and *aph(6)-Id*), and tetracyclines (*tet*(B) observed. Among these genes, *aph(3’’)-Ib*, *aph(6)-Id*, *bla*_ADC-25_, *bla*_OXA-23_, *bla*_OXA-66_, and *tet*(B) were detected in all isolates (Figure 2). The SXT-related resistance genes, *sul1* and *sul2,* had less distribution in CO isolates (45.5%) than in SI isolates (100%), leading to lower MICs. Plasmids were also found to contain no resistance genes except *bla*_OXA-23_, indicating that the co-occurrence of resistance genes on the chromosome and plasmids only occurred with *bla*_OXA-23_.

**Figure 3:**
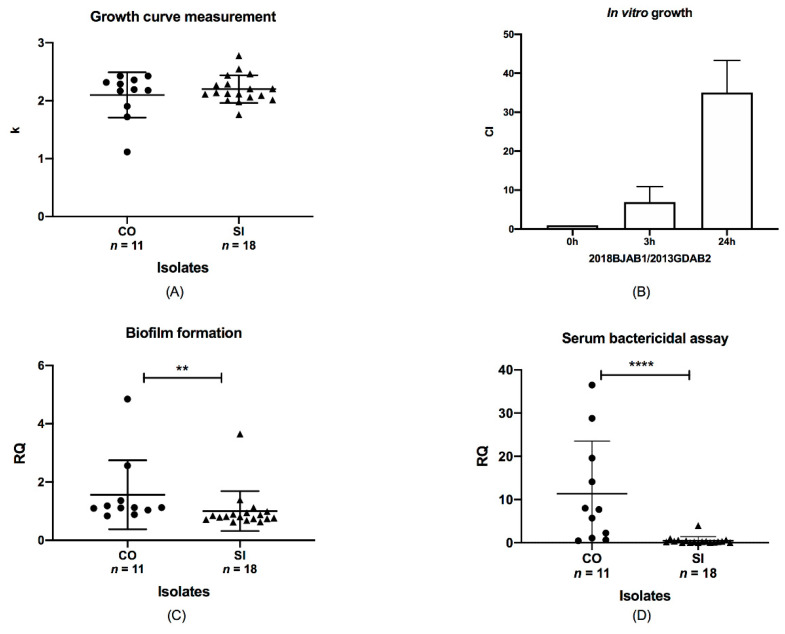
Relationship between phenotype discrepancies and *bla*_OXA-23_ location. Data were analyzed using the Mann–Whitney test, *p* < 0.05. (**A**) Analysis of the growth of CO and SI isolates. The ordinate represented the corrected k value. (**B**) Competition indices of CO and SI isolates at 0, 3, and 24 h. 2018BJAB1, CO isolates; 2013GDAB2, SI isolate. (**C**) Biofilm formation by CO and SI isolates. Two asterisks (**) indicated a significant difference (*p* < 0.01). The ordinate represented the corrected adhesion unit. (**D**) Serum resistance of CO and SI isolates were shown by the survival of all isolates after incubation in normal human serum. Four asterisks (****) indicated a significant difference (*p* < 0.0001). The dots represented CO isolates and the triangles represented SI isolates.

To assess the impact of *bla*_OXA-23_ co-occurrence on chromosome and plasmid on the growth of *A. baumannii* isolates, we compared the growth rates of CO isolates with those of SI isolates. The k value for ATCC19606 was set at 1. However, no growth differences were observed between them (*p* > 0.05, Figure 3A), indicating that the number and position of *bla*_OXA-23_ had little effect on the growth rate. In the context of antibiotic existence (8 mg/L imipenem), the amount of *bla*_OXA-23_ in isolates had little influence on the growth rate (data not shown). In addition, we further investigated in vitro growth competition between CO and SI isolates with the same ST. Paired ST195 CO and SI isolates were co-incubated, and the growth rate of the CO isolate was found to be higher than that of the SI isolate (Figure 3B). We also observed a significant increase in the growth of CO isolates from 3 to 24 h, with the competitive index (CI) increasing from 6.93 to 35.0. The co-occurrence of *bla*_OXA-23_ offered a fitness advantage compared to the SI isolates. The fitness was enhanced by a longer co-incubation time, which suggested that the *bla*_OXA-23_ co-occurrence might be associated with a lower degree of fitness in vitro.

We assessed the biofilm production capacity of SI and CO isolates using a biofilm assay. The results indicated that biofilm production varied between the CO and SI isolates (*p* < 0.01; Figure 3C). The mean values for the CO and SI isolates were 1.56 and 1.002, respectively, and their SD values were 1.185 and 0.6859, respectively. The adhesion unit of biofilm formation for ATCC19606 was set at 1. All isolates produced large quantities of biofilm, but CO isolates produced greater biofilm quantities in general. This significant variation suggests that biofilm formation capacity changing might be related to *bla*_OXA-23_ location diversity. 

We determined the survival of CO and SI isolates after 3 h of incubation in NHS. Half of the CO isolates showed high survival rates of 10% or higher and were characterized as serum resistant. The percent survival for ATCC17978 was set at 100%. SI isolates remained sensitive to serum, with a survival rate of <1%. We examined the results using the Mann–Whitney test and that survival rates were significantly different between CO and SI isolates (*p* < 0.0001; Figure 3D). The ranges of the CO and SI isolates were distinct from 36.05 to 3.983, respectively. The mean survival rates of CO and SI were 11.35 and 0.4902, respectively, and the SD values were 12.21 and 0.9094, respectively. This indicated that *bla*_OXA-23_ co-location on plasmids and chromosomes might correlate with increased serum resistance. Therefore, *bla*_OXA-23_ co-occurrence may be relevant with fitness changes in *A. baumannii* in terms of biofilm formation and serum resistance.

### 2.3. Comparative Analysis of Virulence-Related Genes and Accessory Genome in CO and SI Isolates

**Figure 4:**
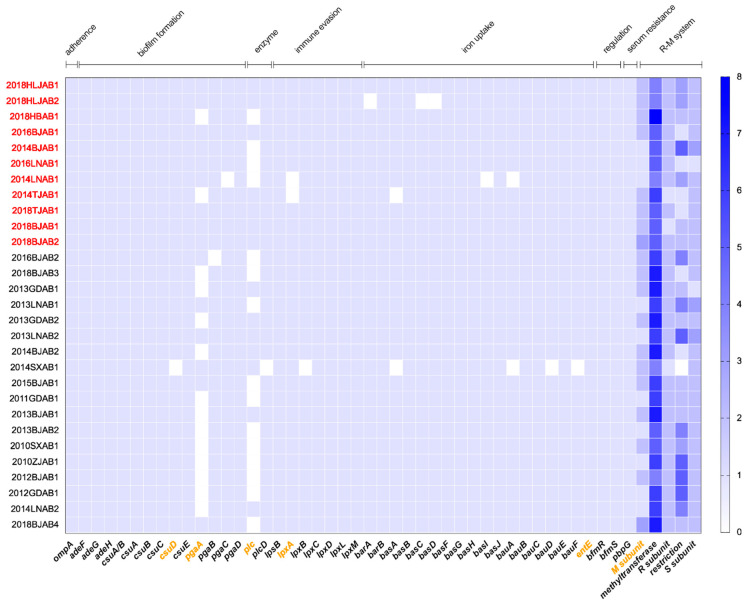
Distribution of virulence and R-M system-related genes in CO and SI isolates. The blue color shade indicated gene presence. The red marked isolates represented CO isolates and the orange marked genes represented differential genes between CO and SI. Virulence gene types were labeled in the upper part.

To investigate differences in virulence capacity between CO and SI isolates, we identified virulence-associated genes using VFDB. The virulence gene profiles showed that most of the virulence genes were present in all isolates, except for the biofilm formation-related genes *csuD* and *pgaA*, the iron uptake-related gene *entE*, the LPS-related gene *lpxA*, and the enzyme-related gene *plc* (Figure 4). The carriage ratios of *csuD* (93.1%), *entE* (93.1%), *pgaA* (81.8%), and *plc* (63.6%) were higher for CO isolates than for SI isolates. Due to the potential loss of defense systems in prokaryotic genomes during evolution, we censored the divergences of restriction-modification (R-M) system-related genes between CO and SI isolates. The R-M systems are an important component of defense systems [12]. BLAST-based on genes from the PADS Arsenal was performed [13], and all isolates were found to contain R-M-encoding genes (Figure 4). Modification subunit (M subunit) encoding genes had a higher distribution in CO isolates, however, all others subunits had a lower distribution.

Moreover, we evaluated discrepancies between CO and SI isolates at the genetic level to determine if there were other genes responsible for the phenotypic changes. Using sequence BLAST and gene absence and presence identification, we compared the presence and absence of genes between CO and SI isolates. Genes and isolates were hierarchically clustered using one minus Pearson correlation metric, with CO and SI isolates clustered separately (Figure S2). We further investigated gene presence and absence in isolates with the same ST (Oxford scheme). Five ST208 SI isolates were found to carry the XRE family transcriptional regulator encoding gene, which was absent in all ST208 CO isolates.

### 2.4. Mechanism of bla_OXA-23_ Multiplication 

To obtain a general view of the mechanism underlying *bla*_OXA-23_ multiplication in *A. baumannii*, we investigated the *bla*_OXA-23_ transposon (Tn) types on the chromosomes and plasmids of each isolate separately (Table 3). The *bla*_OXA-23_ on the chromosome was carried by either Tn*2006* (8, 78.6%) or Tn*2009* (3, 21.4%). Tn*2009*-harboring isolates were limited to ST208. Isolates 2018HLJAB1, 2018HLJAB2, and 2016BJAB1 harbored double copies of *bla*_OXA-23_ on the chromosome, with both copies carried by Tn*2006*. Isolate 2014TJAB1 was the only isolate with *bla*_OXA-23_ carried on different Tn types; the chromosomal and plasmid copies were carried on Tn*2006* and Tn*2009,* respectively. We further investigated the *bla*_OXA-23_ location in SI isolates, aside from Tn*2006* (9, 50.0%) and Tn*2009* (2, 11.1%), *bla*_OXA-23_ was also flanked by IS*Aba1* (7, 38.9%) (Figure 1). We found that none of the plasmid-located *bla*_OXA-23_ from SI were situated at transposon. We also detected IS*Aba1* upstream of the *bla*_OXA-23_ gene in the CO and SI isolates.

Subsequently, we analyzed discrepancies between transposon insertion sites (Table 3). The most common location of *bla*_OXA-23_-containing Tn*2006* in CO isolates was the AbaR4 resistance island (6, 42.9%). Isolates 2018HLJAB1, 2018HLJAB2, and 2016BJAB1 harbored duplicate copies of *bla*_OXA-23_-containing Tn*2006*. One of the Tn*2006* copies was inserted in the antibiotic resistance island AbaR, interrupted with *comM.* The other copy was inserted in the *znuA* promoter region. Furthermore, isolate 2018HBAB1 acquired *bla*_OXA-23_ through Tn*2006* insertion upstream of flavin mononucleotide (FMN) reductase. Isolate 2014TJAB1 was longer and unique, and its insertion position was the same as that of AbaR25.

The insertion sites of *bla*_OXA-23_-containing Tn*2009* in isolates 2014BJAB1, 2016LNAB1, and 2014LNAB1 might be located inside the phage because there were phage-related proteins both upstream and downstream of this region. As previously reported [14], MDR-ZJ06 is one of the most representative isolates harboring Tn*2009-*located *bla*_OXA-23_. The transposon is inserted in a gene cluster involved in the assembly of P pilus, with a 9-bp target site duplication (5′-CAAAAAATT-3′). We found 11 bases (TTCTATCATAGG) inserted in the region between *parA* and *YeeC* in Tn*2009*. In addition, sequence profiles indicated a different direct repeated (DR) sequence from MDR-ZJ06. Tn*2009* in CO isolates was located on the chromosomes and plasmids and was flanked by a 9-bp duplication (5′-AAAATATTT-3′).

### 2.5. Plasmid Characteristics of CO Isolates

**Figure 5:**
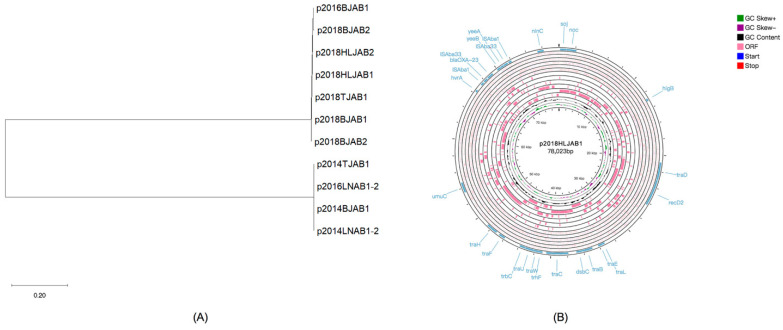
Characteristics of CO plasmids. (**A**) Rooted phylogenetic tree of CO plasmids. (**B**) Circular map of the type II plasmid, p2018HLJAB1. The inner circles represented the GC skew and GC content information. The outer circles represented the open reading frames and the start/ stop codons. Regions related to antibiotic resistance and conjugation were marked in blue.

The evolutionary relationship between *bla*_OXA-23_-harboring CO plasmids was generated through maximum likelihood phylogenetic analysis, in which the plasmids were clustered into two clades (Figure 5A). We also performed pairwise blast analysis of the plasmids to analyze the differences in homology between them, as coverage and identity between plasmids were between 99.84 and 99.99%, and 11%, respectively. Thus, categorizing the 11 plasmids into two groups, CO plasmid type I and II, based on the consequences (Figure S3). The earliest isolate in each group was used as the reference strain to perform circular comparisons of the two types of plasmids, and the overlapping regions of rings confirmed clustering. Plasmids from three 2014 isolates and 2016LNAB1 were more cognate and were classified as CO plasmid type I, which harbored Tn*2009*. Consequently, we investigated the Tn*2009*-harboring plasmids and found that they were significantly similar (99.98%) to the conjugative plasmid pABTJ1, which effectively contributed to the wide dissemination of *bla*_OXA-23_ in *Acinetobacter spp.* in China [15]. 

While the plasmids in all 2018 isolates and 2016BJAB1 were classified as CO plasmid type II and showed substantial similarities, they had different structural characteristics from type I plasmids. Notably, *bla*_OXA-23_ belonged to Tn*2006*, as described earlier. We used BLAST to evaluate the plasmid sequence in NCBI, and no sequences with high homology were found, suggesting that there may have been a new epidemic plasmid carrying *bla*_OXA-23_ in China in 2018. The circular map of p2018HLJAB1, as the representative of the CO type II plasmid, showed that the plasmid was 78,023 bp in size, with 262 open reading frames, and had an average GC content of 33.61% (Figure 5B). The plasmid consisted of two main regions, genes associated with conjugation and *bla*_OXA-23_-containing Tn*2006*. The VFDB analysis confirmed that there were no common virulence-related genes that were present on the CO plasmids. Meanwhile, three genes on the CO plasmids, which include a type II restriction enzyme methylase subunit encoding gene, a type II toxin-antitoxin system RelE/ParE family toxin ParE encoding gene in the CO type II plasmids, and an N-6 DNA methylase encoding gene in both CO type I and II plasmids were associated with virulence according to the clusters of orthologous genes functional categories that were identified using the eggNOG database [16]. Further, we inquired into the *bla*_OXA-23_-carrying plasmids from SI simultaneously and discovered that all three plasmids could be characterized into CO plasmid type I with a few differences in *bla*_OXA-23_ location sites. The genomic environment showed that apart from *bla*_OXA-23_, there were no other resistance genes located in SI plasmids. 

## 3. Discussion

In many countries, including China, the emergence of CRAB-related nosocomial infection outbreaks has been rapidly increasing, leading to treatment failure and prolonged hospitalization [17,18]. As the most representative carbapenem resistance gene, the co-occurrence of *bla*_OXA-23_ on the chromosomes and plasmids may induce phenotypic changes in bacteria. In this study, we describe the carbapenem-resistant *A. baumannii* isolates that *bla*_OXA-23_ co-existed on the chromosome and plasmid for the first time.

Gene amplification is the method most used by bacteria to increase the expression of resistance genes and can, therefore, promote resistance to antibiotics. However, previous studies have shown that *bla*_OXA-23_ multiplication does not enhance carbapenem resistance in clinical CRAB [11]. Our study further proved that the presence of multiple copies and locations of *bla*_OXA-23_ had no significant effect on susceptibility to carbapenems and most antibiotics. However, we found that *bla*_OXA-23_ co-existence had a significant effect on SXT sensitivity. Our results showed that the MICs of SXT in CO isolates were lower than those in SI isolates. In an environment without antibiotics, most resistance mutations reduce bacterial fitness [19]. Based on this premise, *bla*_OXA-23_ amplification and multi-location should have reduced the fitness of the CO isolates. On the contrary, our results suggest that this may be correlated with fitness enhancement. *bla*_OXA-23_ co-occurrence on chromosomes and plasmids altered bacterial phenotypes that are important for bacterial fitness. CO isolates showed better competitive growth, serum tolerance, and biofilm formation capacity, indicating a lower fitness cost in the host and environment. We speculate that this increase in fitness may be concerned with the decreased resistance to SXT, which is related to *bla*_OXA-23_ co-existence. An SXT susceptible *Staphylococcus aureus* isolate was found to produce a lower bacterial load in infected mice than a resistant strain, indicating lower fitness cost [20]. Moreover, plasmids induce the accumulation of both positive and negative effects on bacterial fitness [21]. Plasmids carrying *bla*_OXA-23_ may influence fitness in a positive way. During the evolution of antimicrobial resistance, bacteria may acquire more and differentially located *bla*_OXA-23_ to better adapt to the environment, which implies a greater possibility of extending the host range and increasing spread.

High survival rate in serum and high biofilm formation capacity is closely related to bacterial virulence. This study showed that the CO isolates harbored more virulence genes (including *pgaA* and *lpxA*) than SI isolates. *pgaA* is related to β-1-6-poly-N-acetyl-D-glucosamine (PNAG) synthesis. PNAG is a surface polysaccharide that is indispensable for maintaining biofilm integrity [22]. The increase in *pgaA* in CO isolates may contribute to their higher biofilm production capacity. *lpxA* is a lipid A biosynthesis gene, and lipid A is one of the components of LPS. LPS is critical for in vitro serum resistance and contributes to the survival and fitness of *A. baumannii* [23]. Increased *lpxA* in CO isolates may help bacteria manifest a higher serum tolerance to escape the host’s immune response. The modification subunit genes of the R-M system were different in CO and SI isolates. The R-M system functions by methylating or cleaving DNA to protect bacteria from invasion by foreign DNA, and is also involved in the adaptation of bacteria to changes in environmental conditions [24]. Gene presence-absence identification in ST208 CO and SI isolates revealed variations in an XRE family transcriptional regulator-encoding gene. In a previous study, the first member of the XRE family transcriptional regulator, StrT, was found to be involved in stress tolerance and virulence in *Streptococcus suis* [25]. Further research is needed to clarify the function and mechanisms of R-M system and XRE family transcriptional regulator in *A. baumannii* evolution.

Horizontal gene transfer (HGT), including plasmid conjugation, plays an important role in the dissemination of antibiotic resistance genes, allowing the transfer of genes between isolates and species [26]. Thus, HGT can accelerate the spread of resistance genes and increase global antimicrobial resistance. In conjugative plasmids, resistance genes are usually carried by a transposon, which can transfer genes between plasmids and chromosomes [27]. *bla*_OXA-23_ dissemination is mostly mediated by Tn*2008*, Tn*2006,* and Tn*2009* in *A. baumannii* isolates [6]. Likewise, our results indicate that *bla*_OXA-23_ genes co-located on chromosomes and plasmids were either carried by Tn*2006* or Tn*2009*. Tn*2006* is the most frequently described *bla*_OXA-23_-harboring transposon, and our results support this finding. Tn*2006* has sufficient intercellular transfer efficiency in transferring genes between isolates via plasmids or itself. In contrast, the dissemination of Tn*2009* was attributed to the clonal spread of the bacterial host [28], as Tn*2009*-harboring isolates in our study were limited to ST208. IS*Aba1* belongs to the IS*4* insertion sequences family, which is associated with several antibiotic resistance genes in *A. baumannii* [29]. It plays an important role in *bla*_OXA-23_ gene expression, as the IS*Aba1* transposase is composed of two open reading frames and can produce a functional protein in response to a frameshift during protein translation [30]. We detected IS*Aba1* upstream of the *bla*_OXA-23_ gene and speculate that it might contain promoter sequences for *bla*_OXA-23_ expression; however, future expression studies will be required to fully unravel the influences of the co-occurrence of *bla*_OXA-23_ in chromosomal and on plasmid DNA. The CO plasmids can be classified into two types, where type II plasmids are speculated to be a new epidemic plasmid associated with *bla*_OXA-23_ occurrence in China. Future studies of molecular epidemiology and functional research into new plasmids are needed.

Different transposon insertion sites were identified. ZnuA is a substrate-binding protein of the zinc-uptake ABC transporter and is relevant in bacterial persistence [31]. Transposons with *bla*_OXA-23_ inserted in the ZnuA promoter region may influence gene synthesis, leading to decreased persistence. The *bla*_OXA-23_ gene of isolate 2018HBAB1 was obtained through Tn*2006* insertion upstream of FMN reductase. FMN and the transcriptional regulator, LuxR, remained intact and had no effect on gene function. Furthermore, insertion of Tn*2009* in the phage affected DNA polymerase V (UmuC), which is error-prone and depends on the lesion-bypass replication mechanism [32]. This might also affect upstream hypothetical proteins. Transposon insertion may result in the creation of novel resistance genes at the locus. Therefore, *comM*-AbaR, an antibiotic resistance island, is the preferred hotspot in *A. baumannii* [33]. The findings of this study were consistent with the previous study, which reported that the most common insertion site of Tn*2006* in CO *A. baumannii* strains is located within *comM*-AbaR. The Tn*2006* insertion site in CO isolates was found to be between the coding region of a hypothetical protein and the transcriptional regulator, GntR, and this location may affect transcription. In isolate 2014TJAB1, the different transposon types found on the chromosome and plasmid indicate that the origins of the multiple *bla*_OXA-23_ copies might not be the same. Further studies are needed to clarify the evolutionary mechanism of *bla*_OXA-23_.

The co-occurrence of *bla*_OXA-23_ on chromosomes and plasmids was observed in *Proteus mirabilis* [34]. Two *bla*_OXA-23_ copies were detected*,* one on the chromosome and the other on a plasmid, indicating that *P. mirabilis* may be a reservoir for *bla*_OXA-23_. *bla*_OXA-23_ was also carried on Tn*2006* and inserted in AbaR4. The chromosome, Tn2006 alongside AbaR4, was integrated into the *comM* gene, while the plasmid copy was integrated into the putative colicin gene. *bla*_OXA-23_ amplification in the genome of *P. mirabilis* and *E. coli* increases the transmission risk of bacteria of the *Enterobacteriaceae* family [35]. We observed phenotypic changes related to bacterial fitness in CO isolates. In addition, changes may also increase persistence in the environment, which may be associated with the capacity enhancement of bacteria to develop antimicrobial resistance. Future studies that will analyze the mechanism of *bla*_OXA-23_ co-located on the chromosome and plasmids and interspecies transfer of *bla*_OXA-23_ will be required to fully elucidate its mechanism. 

## 4. Materials and Methods

### 4.1. Bacteria Isolates

A total of 11 CRAB isolates harboring multiple copies of *bla*_OXA-23_ co-occurring on the chromosome and plasmids were selected from 135 *A. baumannii* representative isolates in China between 1999 and 2018, which we referred to as CO isolates. In addition, 18 closely evolutionary related isolates with *bla*_OXA-23_ located on the chromosome or plasmid were selected as controls, which we referred to as SI isolates. The principle for choosing these control isolates was based on single nucleotide variant phylogenetic tree analysis of the core genome and multilocus sequence typing using the Oxford scheme. Detailed information on these isolates is presented in Table 1 and Table S1.

### 4.2. Antimicrobial Susceptibility Testing

Antimicrobial susceptibilities were determined by disk diffusion on Mueller–Hinton agar (Bio-Rad, Marnes-La-Coquette, France) and the minimum inhibitory concentrations (MIC) of colistin and doxycycline were determined using the microbroth dilution method. *E. coli* strain ATCC25922 and *P. aeruginosa* strain ATCC27853 were used as reference control strains, and the aforementioned tests were performed following the Clinical and Laboratory Standards Institute (CLSI) guidelines [36]. Susceptibility was interpreted according to the criteria of the CLSI (M100-S29) [37], except for susceptibility to tigecycline, which was interpreted following the FDA criteria (susceptible: ≤2 mg/L; resistant: ≥8 mg/L) for *Enterobacteriaceae*. 

### 4.3. Genome Sequencing and Analyses

Genomic DNA was extracted using QIAamp DNA Mini Kit (Qiagen, Venlo, Holland) and sequenced using PacBio RS II sequencer (Pacific Biosciences, Menlo Park, CA, USA). *De novo* assembly and resequencing were performed following the hierarchical genome assembly process workflow available in SMRT Analysis v2.3.0 (https://www.pacb.com/products-and-services/analytical-software/smrt-analysis/, access at 2020/03/20) [38]. The long reads were combined with Illumina MiSeq paired-end reads of 300 bp for hybrid assembly using Unicycle [39]. Complete genomes were obtained when only one contig per chromosome/plasmid was used, and this contig could be circularized with overlapping reads of >100 bp at both ends. 

Multilocus sequence typing (MLST) 2.0 server from the Center for Genetic Epidemiology (https://cge.cbs.dtu.dk/services/MLST/, access at 2020/08/10) was utilized to analyze the sequence type [40]. Prokka [41] and Resfinder (https://cge.cbs.dtu.dk/services/ResFinder/, access at 2020/08/10) was used to annotate the contigs and screen for the presence of antibiotic resistance genes. The virulence-associated genes by BLAST were annotated based on the virulence factors database (VFDB, http://www.mgc.ac.cn/VFs/main.htm, access at 2021/08/10) [42]. IS elements were identified using ISfinder (https://isfinder.biotoul.fr/blast.php, access at 2020/08/10) [43]. Restriction-modification (RM) system-related genes were investigated via BLAST using PDAS Arsenal [13]. The plasmids phylogenetic analysis was generated with MEGA X [44]. Plasmids were aligned and visualized using BLAST Ring Image Generator and the CGView server [45,46]. iTOL was used as a manipulation and annotation tool for phylogenetic analysis [47].

### 4.4. Growth Curve Assays

Growth curve assays were performed in triplicate for 29 isolates; 11 CO and 18 SI isolates. Fresh bacterial cultures were grown overnight at 37 °C with shaking at 200 rpm in Luria–Bertani (LB) broth were diluted, separately transferred to 96-well microplates, and cultured as described above. Bacterial growth was determined by measuring the optical density at a wavelength of 600 nm (OD_600_) for 24 h. Growth rates were determined by fitting the growth data to a logistic growth curve using GraphPad Prism8 and Equation (1), where Y^0^ and Y^M^ represent OD_600_ values at time points 0 and M, respectively, and k is a constant calculated automatically by the program. The k value for ATCC19606 was set at 1.

(1) $$Y\;={\;Y}^{M}\;\times {\;Y}^{0}÷\left(\left({\;Y}^{M}\;-{\;Y}^{0}\right)\;\times \;\exp\left(-k\times x\right)\;+{\;Y}^{0}\right)$$

### 4.5. In Vitro Competition Assay

The in vitro competition assay was performed as previously reported [48], with a few modifications. We chose a pair of SXT-susceptible CO and SXT-resistant SI isolates. Isolates cultures incubated separately overnight in LB broth at 37 °C were diluted to 5.0 × 10^5^ colony-forming units (CFU)/mL, pooled together at a 1:1 ratio in LB broth, and co-cultured at 37 °C. At 0, 3, and 24 h, equivalent numbers of blend bacteria were collected, diluted, and plated on Mueller–Hinton plates with and without 16 mg/L SXT, separating SI from CO isolates. CFU numbers were determined after overnight incubation to assess the competitive growth of the CO and SI isolates. The competitive index (CI) was calculated as (CO CFU/SI CFU)/(inoculated CO CFU/inoculated SI CFU).

### 4.6. Biofilm Formation

Biofilm formation assays were performed as previously described [49]. Isolates were removed from the wells, and their optical densities were measured at OD_600_ for growth. For biofilm staining, 150 μL of 10% crystal violet was added to each well and incubated for 30 min. The absorbance of the biofilms was measured at OD_585_. The adhesion unit was determined as OD_585_/OD_600_. The adhesion unit of biofilm formation for ATCC19606 was set at 1.

### 4.7. Serum Bactericidal Assay

Serum bactericidal assays were performed as previously described to determine serum sensitivity [50]. Aliquots of overnight LB broth diluted bacterial cultures in the mid-log growth phase were washed and resuspended in phosphate-buffered saline in triplicate. Then, the suspensions were added to normal human serum (NHS) and incubated at 37 °C for 3 h. Bacteria cultured in each suspension following inoculation and after incubation were serially diluted and plated. The serum bactericidal effect was expressed as percent survival. Survival rates were calculated as the ratio of the CFUs in the suspension of bacteria with and without NHS, and the percent survival for ATCC17978 was set at 100%.

### 4.8. Statistical Analysis

Statistical analyses were performed using GraphPad Prism 8. Comparisons between CO and SI isolates were performed using the two-tailed unpaired Student’s *t*-test, the Chi-square test and the Mann–Whitney test. *p* values <0.05 were considered significant.

## 5. Conclusions

Our results suggest that *bla*_OXA-23_ co-occurrence on chromosome and plasmid does not enhance carbapenem resistance in *A. baumannii* but may result in fitness improvement. The different origins of *bla*_OXA-23_ and the different transposon insertion sites indicate that the co-existence of the resistance gene on chromosomes and plasmids may complicate its dissemination. We speculate that the simultaneous co-occurrence of multiple copies of *bla*_OXA-23_ on chromosomes and plasmids may have a relation with bacterial fitness increase in the host and environment, which will lead to severe conditions in patients, and increase the risk of the emergence and spread of CRAB.

## Figures and Tables

**Table 1:** Characteristics of *A. baumanii* that co-existed *bla*_OXA-23_ in chromosome and plasmid in our research.

Isolate ID	Year of Isolation	Location	Source	STs- Pasteur	STs-Oxford	Copy Number of *bla*_OXA-23_ in Chromosome	Copy Number of *bla*_OXA-23_ in Plasmid *	Plasmid Size (bp)			
2018HLJAB1	2018	China: Ha’erbin, Heilongjiang	pus	2	368	2	1	78,023			
2018HLJAB2	2018	China: Ha’erbin, Heilongjiang	abdominal fluid	2	368	2	1	78,030			
2018HBAB1	2018	China: Wuhan, Hubei	abdominal fluid	2	218	1	1	110,968		78,023 (*bla*_OXA-23_)	
2016BJAB1	2016	China: Beijing	sputum	2	2143	2	1	77,943			
2014BJAB1	2014	China: Beijing	blood	2	208	1	1	77,530			
2016LNAB1	2016	China: Shenyang, Liaoning	cerebrospinal fluid	2	208	1	1	110,967	77,530 (*bla*_OXA-23_)		11,205
2014LNAB1	2014	China: Shenyang, Liaoning	blood	2	208	1	1	110,998	77,537 (*bla*_OXA-23_)		11,194
2014TJAB1	2014	China: Tianjin	blood	2	195	1	1	77,531			
2018TJAB1	2018	China: Tianjin	blood	2	208	1	1	78,022			
2018BJAB1	2018	China: Beijing	sputum	2	195	1	1	78,024			
2018BJAB2	2018	China: Beijing	bronchial	2	195	1	1	78,023			

* The plasmid that possess *bla*_OXA-23_.

**Table 2:** The MIC_50_, MIC_90_, and MIC range (µg/mL) of CO and SI isolates.

Isolates		Imipenem	Meropenem	Ceftazidime	Cefepime	TZP	Colistin	Doxycycline	Minocycline	Tigecycline	Amikacin	Ciprofloxacin	Levofloxacin	SXT
CO *n* = 11	R%	100	100	100	100	100	0	100	81.8	27.3	81.8	100	100	45.5
	S%	0	0	0	0	0	100	0	18.2	72.7	18.2	0	0	54.5
	MIC_50_	64	64	>256	128	>256	0.5	32	16	4	>256	64	16	2
	MIC_90_	64	64	>256	>256	>256	1	32	32	8	>256	128	32	32
	MIC range	64	16~64	64~>256	64~>256	>256	0.25~1	32~32	4~32	2~16	4~>256	64~128	8~32	0.25~64
SI *n* = 18	R%	100	100	94.4	100	100	0	94.4	50	33.3	94.4	100	94.4	88.9
	S%	0	0	5.6	0	0	100	5.6	50	66.7	5.6	0	5.6	11.1
	MIC_50_	64	32	128	64	>256	1	32	8	4	>256	64	16	32
	MIC_90_	64	64	>256	256	>256	1	32	16	8	>256	128	32	128
	MIC range	16~64	16~64	4~>256	32~>256	>256	0.5~2	0.5~64	1~16	1~16	4~>256	16~128	2~64	0.25~128

R, resistance; S, susceptible; MIC_50_, the minimum inhibitory concentrations required to inhibit 50% of isolates; MIC_90_, the minimum inhibitory concentrations required to inhibit 90% of isolates; TZP, piperacillin/tazobactam; SXT, trimethoprim/sulfamethoxazole.

**Table 3:** The distribution of *bla*_OXA-23_-containing transposon and insertion site among the CO isolates.

Isolate	Tn Type of *bla*_OXA-23_		ST (Oxford Scheme)	Tn Insertion Site in Chromosome		Tn Insertion Site in Plasmid	
	Chromosome	Plasmid		Gene Interrupted	Gene Function	Gene Interrupted	Gene Function
2018HLJAB1 *	Tn*2006/* Tn*2006*	Tn*2006*	368	the promoter region of *znuA/ abaR*	substrate-binding protein of zinc uptake ABC transporter/AbaR4	hypothetical protein	unknown
2018HLJAB2 *	Tn*2006/* Tn*2006*	Tn*2006*	368	the promoter region of *znuA/ abaR*	substrate-binding protein of zinc uptake ABC transporter/AbaR4	hypothetical protein	unknown
2016BJAB1 *	Tn*2006/* Tn*2006*	Tn*2006*	2143	the promoter region of *znuA/ abaR*	substrate-binding protein of zinc uptake ABC transporter/AbaR4	hypothetical protein	unknown
2018HBAB1	Tn*2006*	Tn*2006*	218	*FMN* upstream	reductase	hypothetical protein	unknown
2018TJAB1	Tn*2006*	Tn*2006*	208	*abaR*	AbaR4	hypothetical protein	unknown
2018BJAB1	Tn*2006*	Tn*2006*	195	*abaR*	AbaR4	hypothetical protein	unknown
2018BJAB2	Tn*2006*	Tn*2006*	195	*abaR*	AbaR4	hypothetical protein	unknown
2014TJAB1	Tn*2006*	Tn*2009*	195	*abaR*	AbaR25	hypothetical protein	unknown
2014BJAB1	Tn*2009*	Tn*2009*	208	Phage related gene	DNA polymerase	hypothetical protein	unknown
2016LNAB1	Tn*2009*	Tn*2009*	208	Phage related gene	DNA polymerase	hypothetical protein	unknown
2014LNAB1	Tn*2009*	Tn*2009*	208	Phage related gene	DNA polymerase	hypothetical protein	unknown

* The chromosome possessed double copies of *bla*_OXA-23_.

## Data Availability

Data supporting this study are available in the supplementary materials. In addition, Complete genome sequences and raw PacBio sequences were deposited in the National Center for Biotechnology Information (NCBI) database (http://www.ncbi.nlm.nih.gov/, access at 2020/07/16) and available with the BioProject accession number PRJNA646312.

## Supplementary Materials

The following are available online at https://www.mdpi.com/article/10.3390/antibiotics10101196/s1. Figure S1: correlation between antibiotics resistance and *bla*_OXA-23_ location analyzed by Student’s t test, Figure S2: gene presence and absence variants of CO and SI isolates, Figure S3: blast analysis and visualization of *bla*_OXA-23_-carrying plasmids, Table S1: phenotypic and genotypic characteristics of SI *A. baumanii* isolates.
