# Supplementary material for: Co-Occurrence of blaOXA-23 in the Chromosome and Plasmid: Increased Fitness in Carbapenem-Resistant Acinetobacter baumannii
Source: Antibiotics (Basel). 2021 Oct 1;10(10):1196. doi: 10.3390/antibiotics10101196 (PMC8532878; doi:10.3390/antibiotics10101196)
Supplement: Supplementary file 1 [file antibiotics-10-01196-s001.zip › Supplementary Materials.pdf]

Supplementary Materials

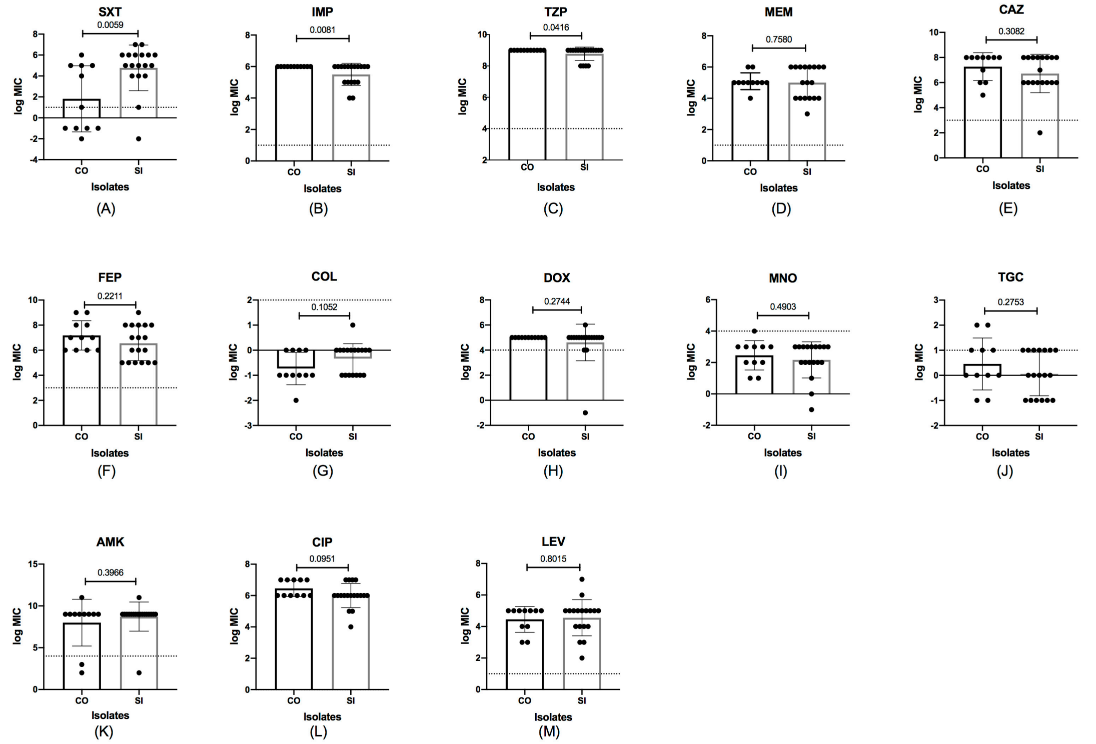

Figure S1: Correlation between antibiotics resistance and *bla*OXA-23 location analyzed by Student's t test. Panels (A) to (M) showed the comparison of the logarithm of MICs of 13 antibiotics to the base of 2 between SI and CO isolates, respectively. If the data possessed unequal standard deviations (SDs), Welch's t tests were executed to correct the variances. The p-value was marked on the figures respectively. P<0.05 specified a significant difference. The dotted lines displayed the logarithm of sensitive breakpoints to the base of 2.

IMP, imipenem; MEM, meropenem; CAZ, ceftazidime; FEP, cefepime; TZP, piperacillin/tazobactam; COL, colistin; DOX, doxycycline; MNO, minocycline; TGC, tigecycline; AMK, amikacin; CIP, ciprofloxacin; LEV, levofloxacin; SXT, trimethoprim/sulfamethoxazole.

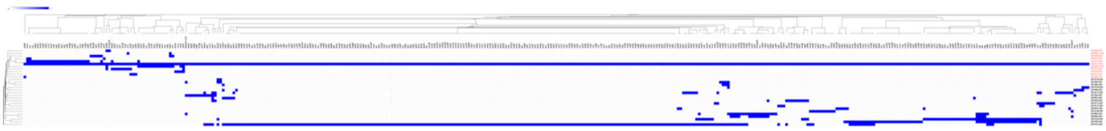

Figure S2: Gene presence and absence variants of CO and SI isolates. The red marked isolates represent CO isolates. The blue color shades indicated presence of the genes.

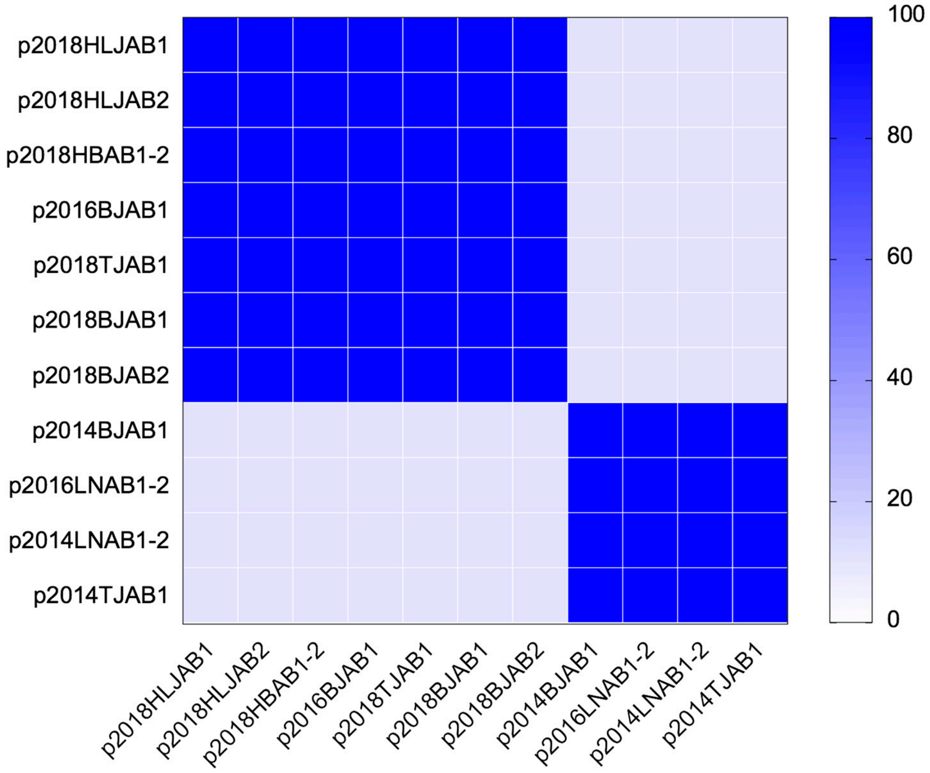

Figure S3: Blast analysis and visualization of *bla*$_{OXA-23}$-carrying plasmids. The blue color shades revealed the coverage and identity of sequence.

Table S1: Phenotypic and genotypic characteristics of SI *A. baumanii* isolates.

	**Characteristic**							**MIC (μg/mL)**												
**Isolate**	**Year of isolation**	**Location**	**Origin/Source**	**STs-Pasteur**	**STs-Oxford**	**Location of *bla*OXA-23**	**Copy number of *bla*OXA-23**	**Imipenem**	**Meropenem**	**Ceftazidime**	**Cefepime**	**TZP**	**Colistin**	**Doxycycline**	**Minocycline**	**Tigecycline**	**Amikacin**	**Ciprofloxacin**	**Levofloxacin**	**SXT**
2016BJAB2	2016	China: Beijing	blood	2	208	plasmid	1	64	64	64	64	>256	1	64	8	2	>256	>32	32	32
2018BJAB3	2018	China: Beijing	blood	2	1968	chromosome	2	64	64	>128	128	>256	1	32	8	2	>256	>64	32	32
2013GDAB1	2013	China: Guangzhou, Guangdong	blood	2	195	chromosome	1	64	64	>128	256	>256	1	32	8	1	>256	>32	32	64
2013LNAB1	2013	China: Shenyang, Liaoning	sputum	2	208	chromosome	1	16	8	64	32	256	0.5	32	8	1	>256	>32	32	32

2013GDAB2	2013	China: Guangzhou, Guangdong	sputum	2	195	chromosome	2	64	64	>128	256	>256	0.5	32	8	2	>256	>32	64	128
2013LNAB2	2013	China: Shenyang, Liaoning	sputum	2	208	chromosome	1	32	16	64	32	>256	0.5	32	8	0.5	>256	>32	16	64
2014BJAB2	2014	China: Beijing	urine	2	195	chromosome	2	32	32	>128	256	>256	1	32	8	2	>256	>64	32	128
2014SXAB1	2014	China: Xi'an, Shanxi	blood	2	195	chromosome	1	>32	64	>128	256	>256	1	32	4	0.5	>256	64	8	32
2015BJAB1	2016	China: Beijing	blood	2	208	plasmid	1	>32	64	128	128	>256	1	32	8	1	>1024	64	16	64
2011GDAB1	2011	China: Guangzhou, Guangdong	sputum	2	457	chromosome	1	16	16	>128	128	256	1	32	4	2	>256	>32	32	0.25
2013BJAB1	2013	China: Beijing	sputum	2	195	chromosome	2	64	64	>128	256	>256	0.5	32	4	2	>256	>32	16	64
2013BJAB2	2013	China: Beijing	blood	570	493	chromosome	1	32	16	64	64	>256	1	0.5	0.5	0.5	>256	>32	>64	2

2010SXAB1	2010	China: Xi'an, Shanxi	sputum	2	1962	plasmid	1	64	64	64	32	256	0.5	16	2	0.5	>256	32	8	16
2010ZJAB1	2010	China: Hangzhou, Zhejiang	blood	2	218	chromosome	1	32	16	64	32	256	1	32	4	0.5	4	32	16	16
2012BJAB1	2012	China: Beijing	blood	2	1962	chromosome	2	>32	16	64	32	>256	1	32	4	1	>256	>64	32	64
2012GDAB1	2012	China: Guangzhou, Guangdong	bronchial	2	208	chromosome	1	>32	32	64	32	>256	0.5	32	8	1	>256	64	32	64
2014LNAB2	2014	China: Shenyang, Liaoning	blood	2	368	chromosome	2	>32	32	4	64	>256	0.5	16	1	0.5	>256	16	4	16
2018BJAB4	2018	China: Beijing	tracheal aspirate	2	1968	chromosome	1	32	16	>128	>256	>256	2	32	4	2	>256	>64	32	32

MIC, minimum inhibitory concentration; TZP, piperacillin/tazobactam; SXT, trimethoprim/sulfamethoxazo.
